# Antigen Uptake After Intradermal Microinjection Depends on Antigen Nature and Formulation, but Not on Injection Depth

**DOI:** 10.3389/falgy.2021.642788

**Published:** 2021-04-08

**Authors:** Romain J. T. Leboux, Pim Schipper, Toni M. M. van Capel, Lily Kong, Koen van der Maaden, Alexander Kros, Wim Jiskoot, Esther C. de Jong, Joke A. Bouwstra

**Affiliations:** ^1^Division of BioTherapeutics, Leiden Academic Centre for Drug Research, Leiden University, Leiden, Netherlands; ^2^Department of Experimental Immunology, Academic Medical Center, Amsterdam, Netherlands; ^3^Division of Supramolecular Chemistry, Leiden Institute of Chemistry, Leiden University, Leiden, Netherlands; ^4^Tongji School of Pharmacy, HuaZhong University of Science and Technology, Wuhan, China; ^5^Tumor Immunology Group, Department of Immunology, Leiden University Medical Center, Leiden, Netherlands; ^6^TECO Development GmbH, Rheinbach, Germany

**Keywords:** liposomes, Bet v 1, intradermal injection, antigen uptake, allergen specific immunotherapy, injection depth, ovalbumin

## Abstract

The skin is an attractive alternative administration route for allergy vaccination, as the skin is rich in dendritic cells (DCs) and is easily accessible. In the skin multiple subsets of DCs with distinct roles reside at different depths. In this study antigen (=allergen for allergy) formulations were injected in *ex vivo* human skin in a depth-controlled manner by using a hollow microneedle injection system. Biopsies were harvested at the injection site, which were then cultured for 72 h. Subsequently, the crawled-out cells were collected from the medium and analyzed with flow cytometry. Intradermal administration of ovalbumin (OVA, model antigen) solution at various depths in the skin did not affect the migration and maturation of DCs. OVA was taken up efficiently by the DCs, and this was not affected by the injection depth. In contrast, Bet v 1, the major allergen in birch pollen allergy, was barely taken up by dermal DCs (dDCs). Antigens were more efficiently taken up by CD14^+^ dDCs than CD1a^+^ dDCs, which in turn were more efficient at taken up antigen than Langerhans cells. Subsequently, both OVA and Bet v 1 were formulated in cationic and anionic liposomes, which altered antigen uptake drastically following intradermal microinjection. While OVA uptake was reduced by formulation in liposomes, Bet v 1 uptake in dDCs was increased by encapsulation in both cationic and anionic liposomes. This highlights the potential use of liposomes as adjuvant in intradermal allergy vaccine delivery. In conclusion, we observed that antigen uptake after intradermal injection was not affected by injection depth, but varied between different antigens and formulation.

## Introduction

Allergen specific immunotherapy through vaccination is the only curative treatment for allergies. These allergy vaccines are traditionally administered subcutaneously (SCIT), but products for sublingual administration (SLIT) are available as well (1). Both therapies are effective, but take 3–5 years to reach effectivity and require an intensive dosing regimen, which contributes to low therapy adherence. To improve therapy adherence, alternative administration sites, and delivery methods are explored (2).

The skin is an interesting administration site for vaccination. It is easily accessible, has a large surface area and a high density of antigen presenting cells (APCs) resides in the skin which allows for a potent immune response (3–6). Previous studies have shown that intradermal vaccination compared to conventional intramuscular or subcutaneous administration can result in equally effective or stronger immune responses, such as rabies (7–9) hepatitis B (10, 11), influenza (12, 13), and polio antigens (14, 15). This illustrates that the intradermal route is an attractive alternative to the conventional vaccination routes.

The main challenge for vaccination via the skin is overcoming the physical barrier, the stratum corneum (3). Several administration methods are available to deliver an antigen (=the allergen in case of allergy) into the skin. One of the most attractive approaches is the use of microneedles, as they are able to bypass the stratum corneum effectively and potentially without pain sensation (16, 17). When the stratum corneum is surpassed, a large network of dendritic cells (DCs) is located in the viable epidermis and dermis. DCs are crucial cells for inducing both humoral and cellular immune responses (18–20).

Several phenotypically and functionally distinct subsets of DCs are known to reside in human skin: Langerhans cells (LCs) are located in the viable epidermis, while CD14^+^, CD1a^+^ dermal DCs (dDCs), and classical DC type 1 cells (cDC1s) are located in the dermis (21). cDC1s are identified by the expression of CD141 and XCR1. These cells are necessary for anti-tumor immunity. They represent a very small fraction of the total skin resident DCs (22).

LCs form a tight network with their dendrites close to the surface of the skin (23–25). Human LCs are recognized by their expression of langerin and a very high expression of CD1a, but lack expression of CD14 on the cell surface. LCs are known to respond to viruses (26–29), but only weakly to bacteria (30–32), probably due to reduced expression of toll-like receptors (TLRs) 2, 4, and 5 (30). Moreover, LCs play a role in skin homeostasis by maintaining a state of tolerance by inhibiting T-cells (33).

CD14^+^ dDCs lack expression of langerin and CD1a, but do express CD14 on the cell surface. It has been suggested that this subset could be monocyte-derived macrophages rather than DCs (34). CD14^+^ dDCs are reported to preferentially polarize naïve CD4^+^ T cells to develop into follicular helper T cells, which in turn induce naïve B cells to produce antibodies and to proliferate into plasma cells (27, 35, 36). CD14^+^ dDCs have shown poor ability to naïve CD8^+^ T-cells (27).

CD1a^+^ dDCs don't have langerin and CD14 expression, but do express CD1a on the cell surface. CD1a^+^ dDCs are intermediately efficient in inducing humoral and cellular immune responses, in comparison to CD14^+^ dDCs and LCs (27, 36–38). Upon activation, LCs and dDCs will take up and process the antigen and subsequently migrate from the skin toward draining lymph nodes, where they present antigen fragments to B- and/or T-cells, initiating the adaptive immune response (23, 24, 39).

Some antigens are poorly taken up by APCs, which complicates the induction of an antigen-specific immune response. Antigen uptake can be increased by formulating antigens in nanoparticles such as liposomes (40–42). Cationic particles are generally taken up more efficiently, as a result of electrostatic interactions with anionic cell surfaces (43, 44). Particles smaller than 500 nm have been shown to be taken up more efficiently by DCs than larger counterparts (45, 46).

The influence of intradermal injection depth on the antigen uptake and subsequent immune response is difficult to examine and so far has not been established. In this study we have used a hollow microneedle based system which allows accurate injection of very small volumes (<1 μL) and controlled injection depth. We set out to obtain fundamental insight in the antigen fate after intradermal microinjection and how formulation into liposomal can alter antigen uptake as well as migration and maturation of LCs and dDCs in a model which directly translates to humans. We compared the uptake of ovalbumin and Bet v 1, the latter is the major allergen responsible for birch pollen allergy. We illustrated that antigen formulation in liposomes can increase uptake in dermal DCs ~10-fold.

## Materials and Methods

### Materials

OVA conjugated with Alexa Fluor® 488, Iscove's Modified Dulbecco's Medium (IMDM), DiI stain, Hank's balanced salt solution and anti-human CD11c-PE-Cy7 antibodies (catalog number 25-0116-42), Alexa Fluor™ 488 NHS ester (succinimidyl ester) were purchased from Thermo Fisher Scientific (Bleiswijk, The Netherlands). Cholecalciferol (vitamin D3) and ethanol 96% (v/v) were obtained from Sigma Aldrich (Zwijndrecht, The Netherlands). Sterile phosphate buffered saline (PBS) (163.9 mM Na^+^, 140.3 mM Cl^−^, 8.7 mM ${\text{HPO}}_{4}^{2-}$, and 1.8 mM H_2_${\text{PO}}_{4}^{-}$, pH 7.4) was ordered at B. Braun Melsungen (Oss, The Netherlands). Skin biopsy punches were purchased from Kai Europe (Solingen, Germany). BD Micro-Fine^™+^ 30G 0.3 mL needle-syringes and anti-human CD1a-APC, CD86-PE, and HLA-DR-PerCP antibodies (catalog numbers 559775, 555665, and 347364, respectively) were obtained from Becton Dickinson (Breda, The Netherlands). Anti-human CD14-APC-Cy7 antibodies (catalog number 301820) were ordered at Biolegend (Koblenz, Germany). HyClone™ fetal calf serum (FCS) was purchased from GE Healthcare Life Sciences (Eindhoven, The Netherlands). GM-CSF was obtained from Schering-Plow (Uden, The Netherlands). Costar® 48-well plates were ordered at Corning Life Sciences (Amsterdam, The Netherlands). Styrofoam was purchased from a local hardware store.

Recombinant Bet v 1 was purchased from the Department of Molecular Biology of the University of Salzburg (Salzburg, Austria). 1,2-dioleoyl-3-trimethylammonium-propane (DOTAP), 1,2-distearoyl-sn-glycero-3-phosphocholine (DSPC), 1,2-distearoyl-sn-glycero-3-phospho-(1'-rac-glycerol) (DSPG) and cholesterol were purchased from Avanti Lipids (Alabama, United States of America). Vivaspin columns (300.000 MWCO) were supplied by Sartorius (Goettingen, Germany).

### Fluorescent Labeling of Antigen

Antigens were labeled according to the manufacturer's protocol (47). In short: proteins were dissolved in 1 mL 100 mM carbonate buffer (pH 8.5) at a concentration of 4 mg per mL (Bet v 1, MW: 17.6 kDa) or 10 mg per mL (OVA, MW: 42.7 kDa). NHS-ester of Alexa Fluor 488 (excitation 490 nm, emission 525 nm) was dissolved in anhydrous DMSO to a concentration of 20 mg/mL. Hundred microliter of the fluorescent dye solution was added to 1 mL protein solution. The mixture was slightly shaken (100 RPM) at room temperature for 1 h in an Eppendorf shaker and subsequently stirred overnight at 4°C. Free dye was removed from protein-bound dye by means of dialysis (2000, Da MWCO) against 10 mM phosphate buffer (pH 7.4). After conjugation, the yield and dye/protein ratio was determined according to the manufacturer's instructions. Protein yield was above 90% in all cases and dye/protein (molar) ratio was between 0.8 and 1.8 and similar for both proteins.

### Preparation of Human Skin for Intradermal (Micro)Injections

Abdominal or breast *ex vivo* human skin was obtained from local hospitals after cosmetic surgery. The procedure was according to the ethical principles of the Declaration of Helsinki. The skin was stored at 4°C and used within 24 h after surgery. The skin surface was cleaned by rinsing it with sterile PBS, 70% (v/v) ethanol and sterile PBS again. The skin was slightly pre-stretched by pinning the skin on a flat piece of Styrofoam in an effort to simulate the stretch conditions of human skin *in vivo*. No difference between abdominal or breast-derived skin was observed, therefore all skin was pooled for analysis.

### Dose-Dependent DC Activation and Antigen Uptake

For each experiment, sterile formulations of OVA and vitamin D3 were prepared. To determine the effect of the administered OVA dose on antigen uptake, migration and maturation of DCs, various doses of OVA in 10 μL PBS were injected intradermally by using a conventional hypodermic needle-and-syringe (30G needle-syringes). As controls, 1.25^*^10^−9^ mole vitamin D3 in 10 μL PBS (positive control) or 10 μL PBS alone (negative control) were injected intradermally. Additionally, plain biopsies of untreated skin were included.

### Depth-Controlled Intradermal Microinjections

To perform injections at an accurate depth, intradermal microinjections were performed by using a digitally-controlled single hollow microneedle injection system (DC-shMN-iSystem). This system comprises a single hollow microneedle, which is fixed in an applicator, as explained in detail elsewhere (48, 49). Accurate intradermal microinjections of very low volumes are feasible by controlling the microneedle applicator and syringe pump (NE-300, Prosense, Oosterhout, The Netherlands) *via* a microneedle applicator controller unit (uPRAX Microsolutions, Delft, The Netherlands) (50, 51). Prior to use, the fluidics part of the DC-shMN-iSystem was sterilized by flushing it with 70% ethanol.

To maximize the accuracy of the microinjection depth, very low volumes were injected to avoid perfusion. Therefore, the microinjection volume was only 0.2 μL and contained 0.1 μg OVA in PBS. Intradermal microinjections were performed at a pre-selected depth of 50, 500, or 1,000 μm by using the DC-shMN-iSystem. As a control, 0.1 μg OVA in 10 μL PBS was injected intradermally with a conventional hypodermic needle-and-syringe (30G needle-syringes).

Liposomes were injected at 500 μm depth in a similar way by using the DC-shMN-iSystem. For all formulations, 0.1 μg of antigen was injected in a volume of 0.2 μL. Consequently, the lipid dose varied between injections and formulations.

### Culturing of Human Skin Explants

Skin biopsies were harvested immediately after intradermal (micro)injection and were cultured as reported earlier (52, 53). A full thickness skin biopsy of 6 mm in diameter was taken from the *ex vivo* human skin with the injection site centrally located. The subcutaneous fat was removed simultaneously. For each treatment, 12 biopsies were harvested. Each biopsy was floated with the epidermal side up for 1 h in 0.5 mL IMDM containing 1% FCS in a 48-well plate. Subsequently, the biopsies were transferred into 1 mL of IMDM containing 10% FCS and 100 ng/mL GM-CSF in a 48-well plate and were cultured with the epidermal side up at 37°C and 5% CO_2_ for 3 days. After removal of the biopsies, migrated cells were harvested and pooled for flow cytometric analysis.

### Preparation of Antigen-Containing Liposomes

Liposomes were prepared according to a dehydration-rehydration method (54). In short: 10 mg lipids were dissolved in chloroform and mixed in the desired ratios (Table 1) with a trace amount (0.2 mol%) of fluorescent lipid Dil (excitation 550 nm, emission 570 nm). The chloroform was removed in a rotary evaporator (150 mbar, 37°C), yielding a lipid film. The lipid film was subsequently hydrated with (antigen-containing) phosphate buffered sucrose (PBS; 10 mM phosphate buffer and 280 mM sucrose, pH 7.4) at 37°C. The lipid-antigen mixture was snap-frozen and lyophilized overnight. The resulting cake was hydrated at 37°C with Milli-Q water in 3 steps: sequentially with 250, 250, and 500 μL, resulting in a suspension containing 10 mg/ml lipids. After each addition, the mixture was briefly vortexed to create a smooth emulsion. The emulsion was homogenized by 6-fold passage over a sequential stack of 400 and 200 nm polycarbonate filter by using an LIPEX extruder (Evonik, Canada). Antigen-loaded liposomes were further purified with centrifuge membrane concentrator (Vivaspin2, 300.000 MWCO, Sartorius), removing the non-associated antigen. All fractions were measured for their fluorescent content (antigen and lipids) in a Tecan Infinite M1000 plate reader (Männedorf, Switzerland) and encapsulation efficiency (EE) was determined as follows:


$$\begin{array}{l} \frac{fluorescence\;after\;purification}{fluorescence\;before\;purification}*100 \end{array}$$

**Table 1:** Liposome formulation composition.

**Formulation name**	**Lipid 1**	**Lipid 2**	**Lipid 3**	**Molar lipid ratio**
Cationic liposomes	DSPC	DOTAP	Cholesterol	2:1:1
Anionic liposomes	DSPC	DSPG	Cholesterol	2:1:1

The hydrodynamic diameter (Z-average), polydispersity index and zeta potential of the liposomes were measured by dynamic light scattering (DLS) and laser Doppler electrophoresis by using a Zetasizer Nano ZS (Malvern Instruments Ltd., Worcestershire, UK). For analysis, each sample was diluted 100-fold in 10 mM sodium phosphate buffer at pH 7.4.

### Analysis by Flow Cytometry

The percentage of specific subsets of total migrated HLA-DR^+^ DCs, maturation and uptake of OVA by migrated LCs, CD1a^+^, and CD14^+^ dDCs were analyzed by using flow cytometry. All migrated cells were isolated and stained with fluorescently-labeled antibodies against CD11c, HLA-DR, CD1a, and CD14. During flow cytometry analysis, migrated DCs were distinguished by their forward and sideward light scattering properties, in combination with high expression levels of HLA-DR and CD11c, after which LCs (defined as CD1a^high^), CD1a^+^ and CD14^+^ dDCs were discriminated from this population (Supplementary Figure 1). Antigen/lipid uptake by LCs, CD1a^+^, and CD14^+^ dDCs was quantified by the percentage of antigen- and/or lipid-containing cells within the LC, CD1a^+^, and CD14^+^ dDC subpopulations. Additionally, maturation of LCs, CD1a^+^ or CD14^+^ dDCs was analyzed by measuring the mean fluorescence intensity (MFI) of the markers HLA-DR and CD86. Multicolor flow cytometry was performed on a FACS Canto II (Becton Dickinson) and FlowJo v10.3 (Tree Star, Ashland, OR, USA) was used for data analysis.

### Statistical Analysis

Graphs were plotted with GraphPad Prism version 8. The data was statistically tested in GraphPad as well, as described in the caption of each figure. To compare antigen uptake between skin donors, uptake was normalized as follows: $\frac{\text{antigen uptake in formulation}}{\text{antigen uptake in PBS}}\times 100\%$. Subsequently, outliers were removed by performing the ROUT outlier test with a 1% false discovery rate.

## Results

### Intradermal Injections Using a Hypodermic Needle and Syringe

Currently intradermal injections are administered with a hypodermic needle and syringe-based system. To evaluate the delivery of antigen after intradermal injection, we have performed a series of experiments using conventional intradermal injections.

#### Three Distinct Dendritic Cell Subsets Migrated of *ex vivo* Human Skin

**Figure 1:**
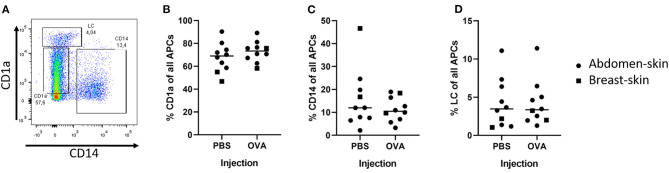
Detection of DCs after intradermal injection of 10 μL with a hypodermic needle and syringe. The migrated DCs were collected from human *ex vivo* skin (circles = abdominal, squares = breast) explants after 72 h of culturing. DCs were defined by expression of both HLA-DR and CD11c. A representative plot of the DCs shows the gating **(A)**. In the DC population 3 subsets were identified: CD1a^+^ dDCs **(B)**, CD14^+^ dDCs **(C)**, and LCs **(D)** (*n* = 10 independent experiments) showing each experiment and mean.

To determine what cells migrate from a skin explant, we injected model antigen OVA and PBS in *ex vivo* human skin and cultured skin biopsies for 72 h. After intradermal injection three DC subsets had migrated out of the skin explant (Figure 1A): LCs (CD14^−^ and CD1a^++^), CD14^+^ dDCs (CD1a^−^ and CD14^+^), CD1a^+^ dDCs (CD1a^+^ and CD14^−^). The majority (ca. 70%) of DCs, as defined by HLA-DR and CD11c expression, were CD1a^+^ dDCs (Figure 1B), 10% were CD14^+^ (Figure 1C) and 4% were LCs (Figure 1D). The percentage of each subset that migrated out of the explant was independent of the injected formulation (PBS vs. OVA), but varied between the donors (Figure 1). Moreover, when skin was not injected at all, similar percentages of cells migrated from the skin explant (not shown). The total amount of cells that crawled out however, was consistently higher after injection of formulations that contained antigen than after PBS injection (data not shown).

#### OVA Uptake by Skin-Resident APCs Is Dose-Dependent

**Figure 2:**
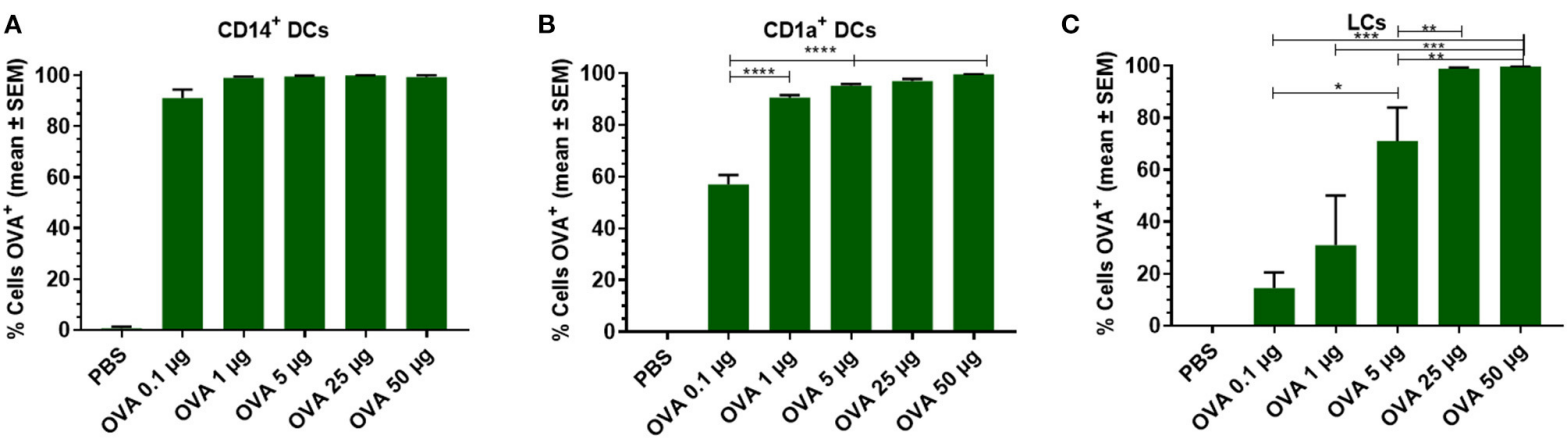
Dose dependency of OVA uptake is DC subset specific. OVA uptake by LCs, CD1a^+^, and CD14^+^ dDCs was investigated by flow cytometry as function of the OVA dose after intradermal administration in abdominal skin by using a conventional hypodermic needle-and-syringe. OVA uptake was measured and displayed as percentage of cells that had taken up OVA within migrated CD14^+^ dDCs **(A)**, CD1a^+^ dDCs **(B)**, and LCs **(C)**. The data represents mean ± SEM (*n* ≥ 3 independent experiments) uptake was compared by one-way ANOVA with Tukey's multiple comparison test. *p < 0.05, **p < 0.01, ***p < 0.001, ****p < 0.0001.

To analyze the effect of the antigen dose on uptake by skin DCs, increasing doses of OVA were injected into the skin and migrated cells were analyzed. Antigen uptake by dDCs and LCs was evaluated after injecting various doses of OVA. Practically all CD14^+^ dDCs had taken up detectable amounts of OVA, even when the low dose of 0.1 μg OVA was administered (Figure 2A). In contrast, ~60% of the CD1a^+^ dDCs had taken up detectable OVA after 0.1 μg OVA was administered (Figure 2B). At higher doses, the majority (>95%) of CD1a^+^ dDCs had taken up OVA. LCs showed a dose-dependent uptake of OVA in the investigated concentration range. Only after administration of the highest doses (50 or 25 μg) all LCs showed detectable amounts of OVA. 70% of LCs had taken up OVA after a 5 μg dose, with a dose-dependent decrease after administration of 1 and 0.1 μg OVA (Figure 2C).

### Depth-Controlled Microinjections via a Hollow Microneedle

Our previous results demonstrated that intradermal delivery of OVA results in uptake by dDCs and LCs. Because the different DC subsets reside at different depths in the skin, we hypothesized that OVA uptake may also depend on the depth of intradermal antigen application. To administer antigen at a specific depth, conventional intradermal injection with needle and syringe cannot be used. Besides the difficulty to determine injection depth accurately, an injection volume of 10 μL (equals 10 mm3) is a too large volume for accurate injection in micrometer ranges. A digitally-controlled single hollow microneedle injection system allows for precise injections of 200 nL, enabling depth-controlled injections of a 50-fold smaller volume.

#### OVA Uptake by Skin-Resident APCs Is Independent of Injection Depth

**Figure 3:**
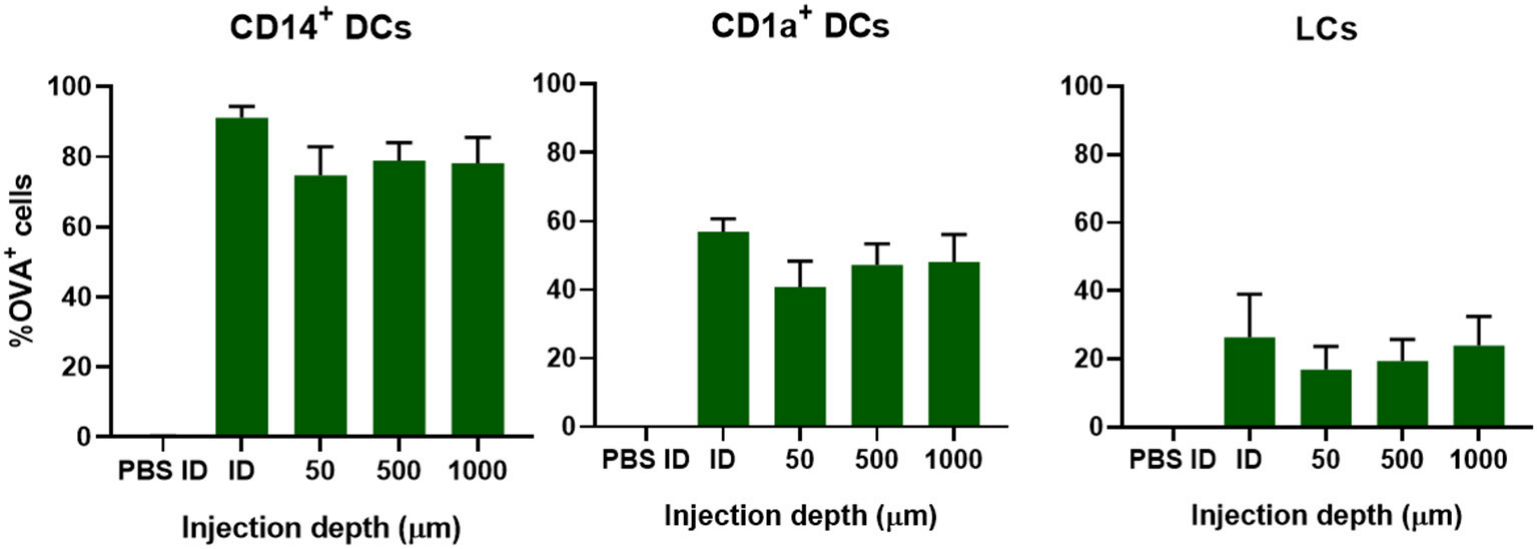
Uptake of OVA by skin-resident DCs is not dependent on injection depth. The uptake of OVA in CD14^+^ dDCs, CD1a^+^ dDCs, or LCs as function of the depth in abdominal skin at which 0.1 μg OVA was administered by using a single hollow microneedle or conventional hypodermic needle-and-syringe (ID). The data represents mean ± SEM (*n* ≥ 4 independent experiments). No statistical differences were found between the different depths (one-way ANOVA).

To study if injection depth affects the uptake of OVA in different subsets of DCs, OVA solution was injected at a depth of 50 μm (viable epidermis), 500 μm (superficial dermis), or 1,000 μm (deep dermis) with a hollow microneedle. This was compared to injections of 0.1 μg OVA with a hypodermic needle, where the injection depth is not known and the injected volume is much bigger: 10 μL instead of 0.2 μL. Regardless of injection depth, 75% of all CD14^+^ dDCs had taken up OVA after injection with hollow microneedles (Figure 3). Conventional intradermal injection resulted in a higher percentage of OVA positive (OVA^+^) cells. Uptake of OVA by CD1a^+^ dDCs and LCs was slightly, albeit not significantly, lower at more shallow (50 μm) depth-controlled injections. Moreover, conventional injection resulted in slightly more OVA^+^ cells in both subsets. Concomitantly, DC activation, as measured by CD86 and HLA-DR expression, was similar for all injection depths and conventional intradermal administration (Supplementary Figure 1). Altogether, injection depth did not influence antigen uptake or DC activation. Therefore, it was decided to perform all subsequent injections at only one depth: 500 μm.

#### Bet v 1 Is Barely Taken Up by Dermal APCs

**Figure 4:**
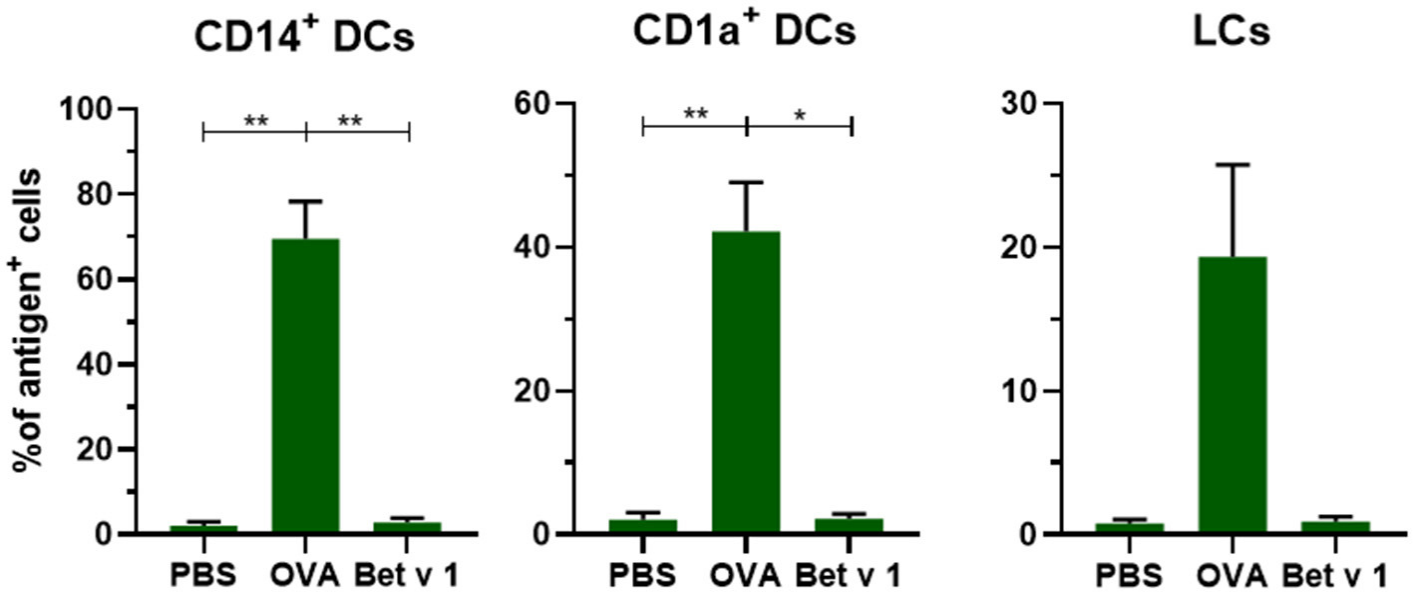
Not all antigens are taken up to the same extent by APCs. The uptake of fluorescent OVA or Bet v 1 in CD14^+^ dDCs, CD1a^+^ dDCs, and LCs after microinjection at 500 μm depth with 0.1 μg of antigen in abdominal skin (mean ± SEM; *n* ≥ 6 independent experiments). The percentage of cells that had taken up antigen was compared in a mixed-effects analysis with a Tukey's multiple comparison test. *p < 0.05, **p < 0.01.

To evaluate whether antigens with different properties are taken up in a similar fashion, we compared the uptake of model allergen OVA (42.7 kDa, pI 4.5) with the uptake of real allergen Bet v 1 (17.4 kD, pI 5.6) after administration of 0.2 μL solution containing 0.1 μg at 500 μm depth. In contrast to OVA, Bet v 1 was barely taken up by any of the migrated dDCs and LCs (Figure 4). The mean fluorescence intensity (MFI) of the injected antigen in CD14^+^ dDCs was slightly increased after microinjection of Bet v 1, but not in the other subsets (Supplementary Figure 1), while the MFI after OVA injection increased significantly. This illustrates that not all intradermally administered protein antigens, when free in solution, are taken up efficiently by APCs.

#### Both Antigens Were Incorporated in Fluorescently Labeled Cationic and Anionic Liposomes

Nanoparticles such as liposomes are generally considered to be efficiently taken up by APCs (55, 56). Nanoparticle mediated uptake could overcome structural differences between antigens. Both OVA and Bet v 1 (size graph in Supplementary Figure 1) were encapsulated in two types of liposomes: cationic and anionic liposomes (Table 2). Cationic liposomes with OVA encapsulated were larger than empty cationic liposomes, but the zeta potential was unchanged. Encapsulation of Bet v 1 in cationic liposomes did not affect the size, but decreased the zeta potential slightly. Anionic liposomes were slightly smaller after encapsulation of both antigens: 180 nm instead of 205 nm, while zeta potential remained negative. The encapsulation efficiency of OVA in cationic liposomes was higher (70%) than that of Bet v 1 (50%). Both OVA and Bet v 1 did not associate well with anionic liposomes (5 and 10%, respectively). The amount of antigen was kept constant for intradermal injections, so the injected dose of lipids was higher for anionic liposomes than for cationic liposomes.

**Table 2:** Physicochemical properties of the liposomal formulations (mean values ± SD, *n* = 3–5).

**Formulation**	**Z-ave (nm)**	**PDI**	**ZP (mV)**	**EE (%)**
Cationic liposomes (DSPC:DOTAP:chol)	184.6 ± 5.6	0.102 ± 0.018	36.0 ± 14.5	–
Anionic liposomes (DSPC:DSPG:chol)	204.9 ± 13.6	0.128 ± 0.004	−44.4 ± 0.5	–
Cationic liposomes containing OVA	206.6 ± 25.1	0.140 ± 0.086	37.1 ± 3.9	70.9 ± 9.2
Anionic liposomes containing OVA	178.0 ± 12.1	0.035 ± 0.018	−38.8 ± 1.8	5.2 ± 0.7
Cationic liposomes containing Bet v 1	194.8 ± 14.9	0.129 ± 0.017	26.0 ± 7.4	49.4 ± 28.3
Anionic liposomes containing Bet v 1	180.6 ± 2.1	0.101 ± 0.023	−47.0 ± 18.8	10.6 ± 5.2

*Z-ave, hydrodynamic diameter; PDI, polydispersity index; ZP, zeta potential; EE, encapsulation efficiency; –, not applicable*.

#### Incorporation of Bet v 1, but Not OVA, in Liposomes Increased Uptake by dDCs

**Figure 5:**
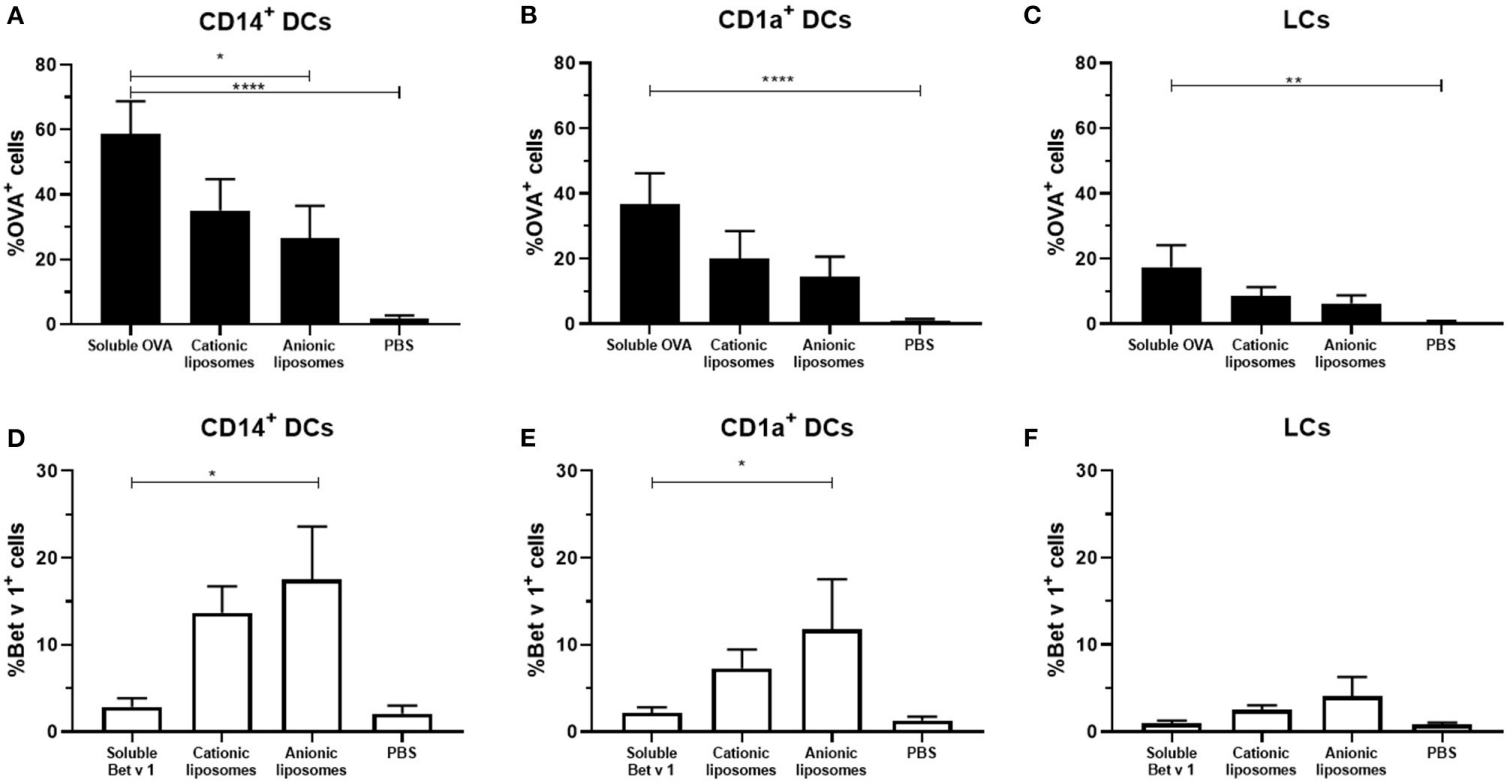
Effect of fluorescently labeled liposomes on antigen uptake. The uptake of fluorescent OVA **(A–C)** and Bet v 1 **(D–F)** in different dDCs or LCs was measured (mean ± SEM; *n* ≥ 6 independent experiments). Antigens (0.1 μg) were injected at 500 μm depth in abdominal (*n* ≥ 4) or breast (*n* = 2) skin explants. The percentage of dDCs that had taken up antigen was compared in a mixed-effects analysis and Dunnett post-test to compare uptake to free antigen. *p < 0.05, **p < 0.01, ****p < 0.0001.

To evaluate the effect of liposome formulation on the uptake of OVA and Bet v 1, formulations were injected in *ex vivo* human skin with hollow microneedles at 500 μm depth. After antigen encapsulation in liposomes an increase in uptake was observed for Bet v 1, but a decrease in uptake was observed for OVA (Figure 5). Encapsulation in cationic liposomes decreased OVA uptake in CD14^+^ dDCs from 60% of all cells (for OVA solution) to 35%, whereas encapsulation of OVA in anionic liposomes decreased its uptake to 25% (Figure 5A). Similar trends were observed for CD1a^+^ dDCs (Figure 5B) and LCs (Figure 5C).

For Bet v 1, an opposite effect was observed. Cationic liposomes increased the percentage of Bet v 1^+^ CD14^+^ dDCs to 14%, while anionic liposomes resulted in 17% antigen^+^ CD14^+^ dDCs (Figure 5D). Bet v 1^+^ CD1a^+^ dDCs percentage increased from 2 to 6% with cationic liposomes, and 12% with anionic liposomes (Figure 5E). A similar trend was observed in LCs, where the Bet v 1^+^ % of cells was increased from 1 to 2.5% and 4%, respectively.

**Figure 6:**
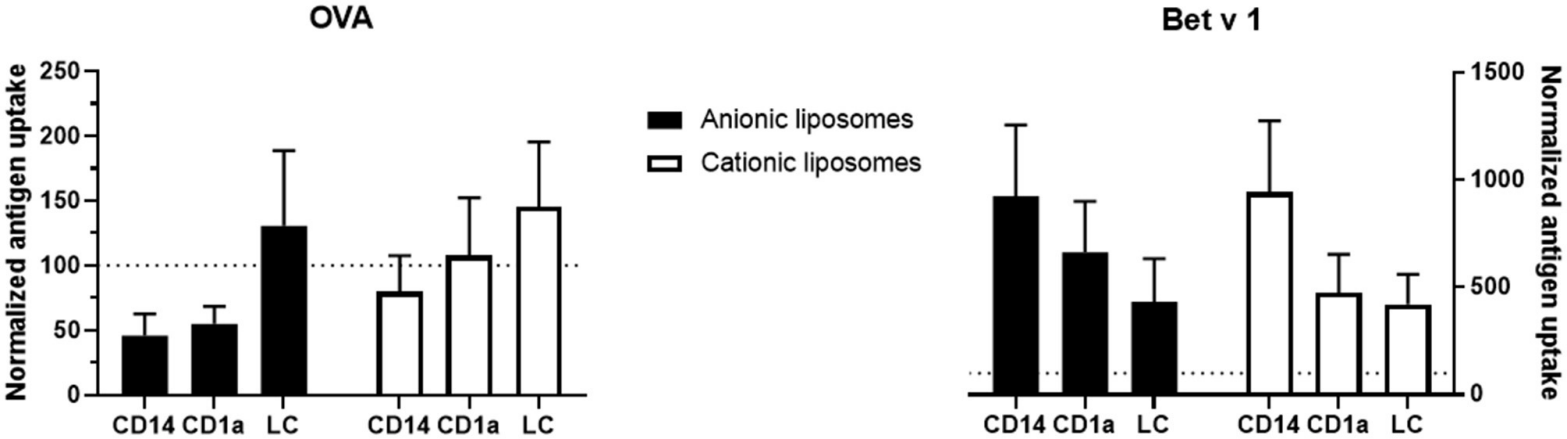
Normalized effect of fluorescently labeled liposomes on antigen uptake. The percentage of cells that had taken up free antigen, as displayed in Figure 5, was set at 100% (dotted line in all graphs). Percentage of cells that had taken up antigen after formulation into liposomes was normalized to the uptake of free antigen. The normalized uptake of both OVA (left) and Bet v 1 (right) incorporated in cationic liposomes (white) and anionic liposomes (black) after administration at 500 μm depth in abdominal (*n* ≥ 4) or breast (*n* = 2) skin explants is shown (mean ± SEM; *n* ≥ 6 independent experiments). Significant differences between dendritic cell subset and formulation were not found in a 2-way ANOVA for either antigen.

Antigen uptake of the same formulation varied substantially between skin donors. Therefore, to evaluate the effect of liposome formulation on antigen uptake, the uptake was normalized, i.e., uptake of free antigen was set as 100% uptake, and compared to uptake of antigen formulated in liposomes per donor (Figure 6). Cationic liposomes had little effect on OVA uptake: uptake in CD14^+^ dDCs was reduced 0.8-fold, whereas uptake in CD1a^+^ dDCs was unaffected and uptake in LCs was increased 1.4-fold (Figure 6). Anionic liposomes reduced uptake in CD14^+^ dDCs and CD1a^+^ dDCs by half, but increased uptake in LCs by 1.3-fold (Figure 6).

Cationic liposomes increased Bet v 1 uptake by CD14^+^ dDCs over 9-fold, while increasing uptake in both CD1a^+^ dDCs and LCs 4.5-fold (Figure 6). Anionic liposomes similarly increased Bet v 1 uptake in CD14^+^ dDCs 9-fold, uptake in CD1a^+^ dDCs 6.6-fold and uptake in LCs 4.3-fold (Figure 6). Uptake of Bet v 1 was increased drastically in especially CD14^+^ and CD1a^+^ dDCs by encapsulation in liposomes. Contrarily, the uptake of OVA was reduced in these subsets after encapsulation in either cationic or anionic liposomes.

#### Anionic Liposomes Are Taken Up More Efficiently Than Cationic Liposomes

**Figure 7:**
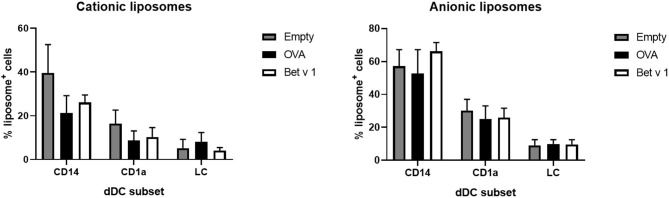
Liposome uptake by skin resident APCs. The uptake of fluorescently labeled cationic liposomes (left) and anionic liposomes (right) in different skin APCs was measured (mean ± SEM; *n* ≥ six independent experiments) after injection at 500 μm depth in abdominal (*n* ≥ 4) or breast (*n* = 2) skin explants. The percentage of dDCs or LCs that had taken up liposomes was compared in a 2-way ANOVA and showed no significant differences between OVA or Bet v 1 in each subset.

To determine whether the cells had also taken up liposomes, both liposome formulations contained a small amount of fluorescent lipid. Liposome uptake by the various subsets of DCs was measured. The loading of antigen did not affect the uptake of liposomes by the various subsets (Figure 7). Cationic liposomes were taken up less efficiently than anionic liposomes. Moreover, antigen formulated with cationic liposomes resulted in lower uptake of liposomes and antigen by the same cell than formulation in anionic liposomes (Supplementary Figure 1). Representative flow cytometry dot plots of each subset and each formulation are shown in Supplementary Figures 6, 7.

## Discussion

In this study we set out to obtain fundamental knowledge about the fate of antigen formulations after intradermal injections. This knowledge is important for rational development of formulations for intradermal administration. We evaluated migrated DCs after intradermal microinjections of two antigens, OVA and Bet v 1, in a human skin explant model. Both antigens were formulated in buffer and in 2 different liposome formulations. Injection depth did not affect antigen uptake, but we observed a significant difference in uptake between antigens. When formulated in buffer, Bet v 1 was barely taken up, whereas OVA was taken up very efficiently by APCs. By incorporating OVA, which was taken up efficiently on its own, lower percentages of APCs had taken up antigen encapsulated in liposomes. On the contrary, Bet v 1 (a relevant, but poorly internalized antigen) was delivered much more efficiently to the dDCs and LCs when encapsulated in liposomes upon injection.

Uptake of antigen upon conventional intradermal injection was dose-dependent. This illustrates the effect the antigen dose could have on the induced immunity. CD14^+^ dDCs are poor at inducing CTLs, but induce a strong humoral response, while LCs are associated with strong CTL responses (27). As these subsets reside at different depths of the skin, we performed injections at different depths to see if injection depth has any effect on uptake in different subsets.

Intradermal microinjections performed at 50, 500, or 1,000 μm did not show any significant difference in antigen fate, even though the different DC subsets reside in different parts of the skin. When compared to intradermal injection with a hypodermic needle, we saw less uptake after depth controlled microinjections. This may be as a result of injection accuracy: the DC-shMN-iSystem allows very precise injection volumes, whereas a conventional needle and syringe based system does not. Besides antigen uptake, DC activation was not affected by the injection depth either. This would also suggest that there will be no injection depth-dependent effect on the immune response. These findings are in corroboration with various vaccination studies in other species, even though skin composition and morphology differs between different species (57, 58). Our results can explain why injection depth did not affect immune response in rats vaccinated with inactivated polio vaccine (48) and hairless guinea pigs vaccinated with OVA by others (59). Moreover, a study on intradermal vaccination of human volunteers with rabies vaccine did not show an injection depth-dependent immune response either (60).

Unlike injection depth, the nature of the antigen had a huge impact on its uptake by DCs. The differences between OVA and Bet v 1 are numerous: OVA is glycosylated and phosphorylated (61), while Bet v 1 does not have such post-translational modifications, as it was produced in *E.coli*. OVA is 3 times heavier than Bet v 1 and has a lower isoelectric point, although both proteins are negatively charged at physiological pH. Especially the post-translational modifications can impact the uptake of a protein (62): the mannose receptor has been shown to play a huge role in OVA uptake (63), while Bet v 1 uptake is reported to be caveolae-mediated (64).

OVA was taken up readily by the majority of CD14^+^ dDCs. Bet v 1, however, was not taken up so easily: <5% of all dDCs and LCs had taken up Bet v 1. The difference in uptake between the two antigens was surprising, as a large number of publications have shown that both antigens are readily taken up in cell culture conditions by human monocytes (21, 36, 64–68). This shows the limitations of cell culture experiments, where cells are continuously exposed to antigen. Thus, there is a translational gap between cell culture and injection in human skin. Our presented *ex vivo* human skin model is more representative for what would happen after intradermal injection.

Two different liposomal formulations were prepared, both having sizes smaller than 500 nm, which has been reported to be ideal for uptake by DCs (45, 46). Both formulations consisted for 50% of DSPC (T_m_ ~55°C) and contained 25% cholesterol. The only difference between the liposome formulations is the charged lipid, which allows for a direct comparison of surface charge effect. DOTAP-containing liposomes were cationic, and had a higher encapsulation efficiency with OVA and bet v 1 than anionic DSPG-containing liposomes. This is most likely related to electrostatic interactions, as both proteins have a negative charge at physiological pH. However, part of the antigens may be associated on the surface of liposomes rather than be encapsulated in the liposome core. This could result in quick desorption after injection, which we indeed seemed to observe: for cationic liposomes there were more OVA^+^ than liposome^+^ cells, which would otherwise not be possible. Free OVA was taken up in more cells (%-wise) than when encapsulated in liposomes, while the uptake of Bet v 1 was increased drastically when encapsulated in liposomes compared to free Bet v 1. This difference can probably be attributed to the uptake of soluble antigen. Encapsulated OVA uptake depends on the uptake of liposomes, which was not as effective as that of soluble OVA.

Formulation of antigens (=allergen in case of allergy) in liposomes could also contribute to more efficient allergen specific immunotherapy. By encapsulating Bet v 1 in the core, it is not available on the surface and cannot bind circulating antibodies and thereby reduce adverse events (2). For this purpose anionic liposomes would be the preferential choice, as there seems to be more antigen dissociation from cationic liposomes. Moreover, by increasing the antigen uptake the effectivity can potentially be increased. The increased effectivity could lead to a reduction of therapy duration (1, 2, 69).

Intradermal vaccination has been reported to induce a stronger CD8^+^ T-cell immune responses compared to conventional subcutaneous or intramuscular injections (70, 71). Most nanoparticle-based approaches use cationic delivery systems, because cationic formulations are taken up to a higher extent than anionic ones *in vitro* (57), and we have seen the same with the formulations we have used (data not shown). Those studies however describe *in vitro* situations, where the extracellular matrix and presence of other cell types (e.g., keratinocytes) is not taken into consideration, which has been shown to reduce delivery of cargo from cationic nanoparticles before (72). We demonstrated that anionic liposomes resulted in more efficient delivery of Bet v 1 to APCs than cationic liposomes in intact human skin. There does not seem to be a targeting effect to any of the subsets. The same uptake pattern (CD14^+^ dDCs > CD1a^+^ dDCs > LCs) is observed with liposomes as for OVA in buffer.

Unexpectedly, anionic liposomes resulted in higher Bet v 1 uptake than cationic liposomes, which have been used successfully in a multitude of intradermal vaccine delivery studies (50, 54, 73, 74). We should however realize that, as antigen dose was kept constant, more liposomes were injected for anionic liposomes than cationic liposomes. Uptake by skin DCs is only the first step in the induction of antigen-specific immunity. The activation state, antigen processing and subsequent T-cell stimulation has not been investigated. Cationic liposomes typically induce an inflammatory Th1 and CD8^+^ T-cell based immune response, which is desired for cancer immunotherapy. Contrarily, anionic liposomes are reported to induce regulatory responses, which could be beneficial for the treatment of allergy or auto-immune diseases (44, 55, 75–79). So, both cationic and anionic liposomes are interesting adjuvant candidates that can increase the uptake of antigens which are not efficiently taken up by themselves.

## Conclusion

Intradermal injection depth of antigens in *ex vivo* human skin does not affect antigen uptake by migrated dDCs and LCs. However, a large difference in effect occurs based on the kind of antigen and the kind of formulation applied. OVA was readily taken up by dDCs and LCs in contrast to Bet v 1, a relevant antigen in allergy. After incorporation in cationic and especially anionic liposomes, Bet v 1 was taken up by more dDCs and LCs. We conclude that both antigen nature and formulation, but not injection depth determine the degree to which antigens are taken up by skin resident APCs. Moreover, we have shown that uptake of poorly internalized antigens can be significantly improved by encapsulating them in liposomes in an *ex vivo* human skin model.

## Data Availability Statement

The original contributions presented in the study are included in the article/Supplementary Material, further inquiries can be directed to the corresponding author/s.

## Author Contributions

RL, PS, LK, TvC, and KvdM contributed to the experimental work. RL, KvdM, WJ, JB, AK, and EdJ wrote the manuscript. Data analysis and processing was performed by RL and PS. All authors contributed to the article and approved the submitted version.

## Conflict of Interest

KvdM was employed by the company uPRAX. WJ is a scientific advisor at Coriolis Pharma, Martinsried, Germany. The remaining authors declare that the research was conducted in the absence of any commercial or financial relationships that could be construed as a potential conflict of interest.
