# Supplementary material for: Antigen Uptake After Intradermal Microinjection Depends on Antigen Nature and Formulation, but Not on Injection Depth
Source: Front Allergy. 2021 Apr 8;2:642788. doi: 10.3389/falgy.2021.642788 (PMC8974696; doi:10.3389/falgy.2021.642788)
Supplement: Supplementary file 1 [file Data_Sheet_1.docx]

# Supplementary information


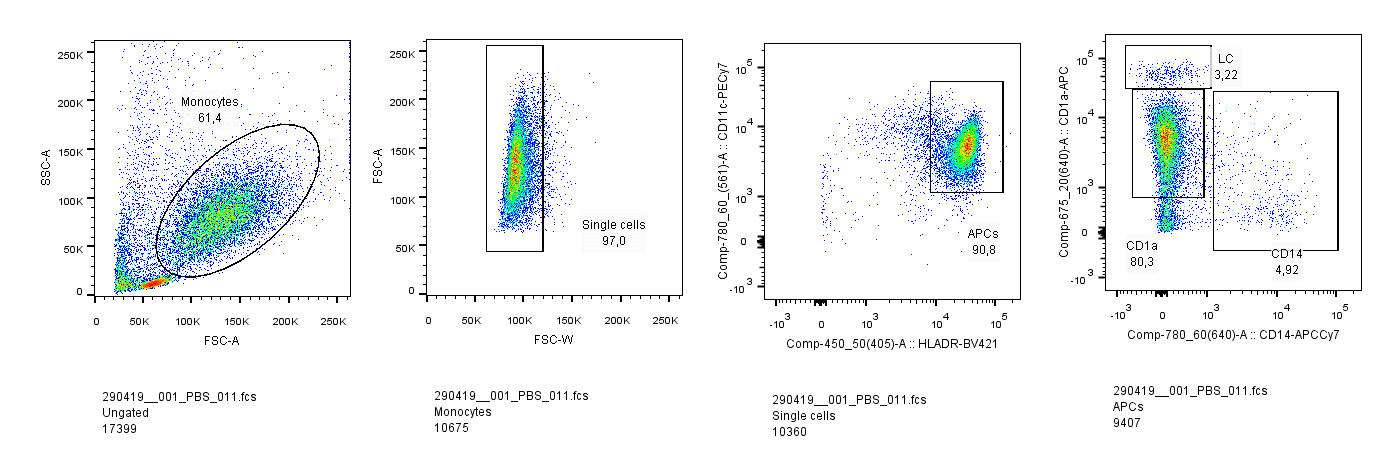


Supplementary Figure 1. Gating strategy for flow cytometry analysis to distinguish the different dermal dendritic cells and LCs. Langerhans cells (LCs), CD1a^+^ dDCs and CD14^+^ dDCs are distinct populations.


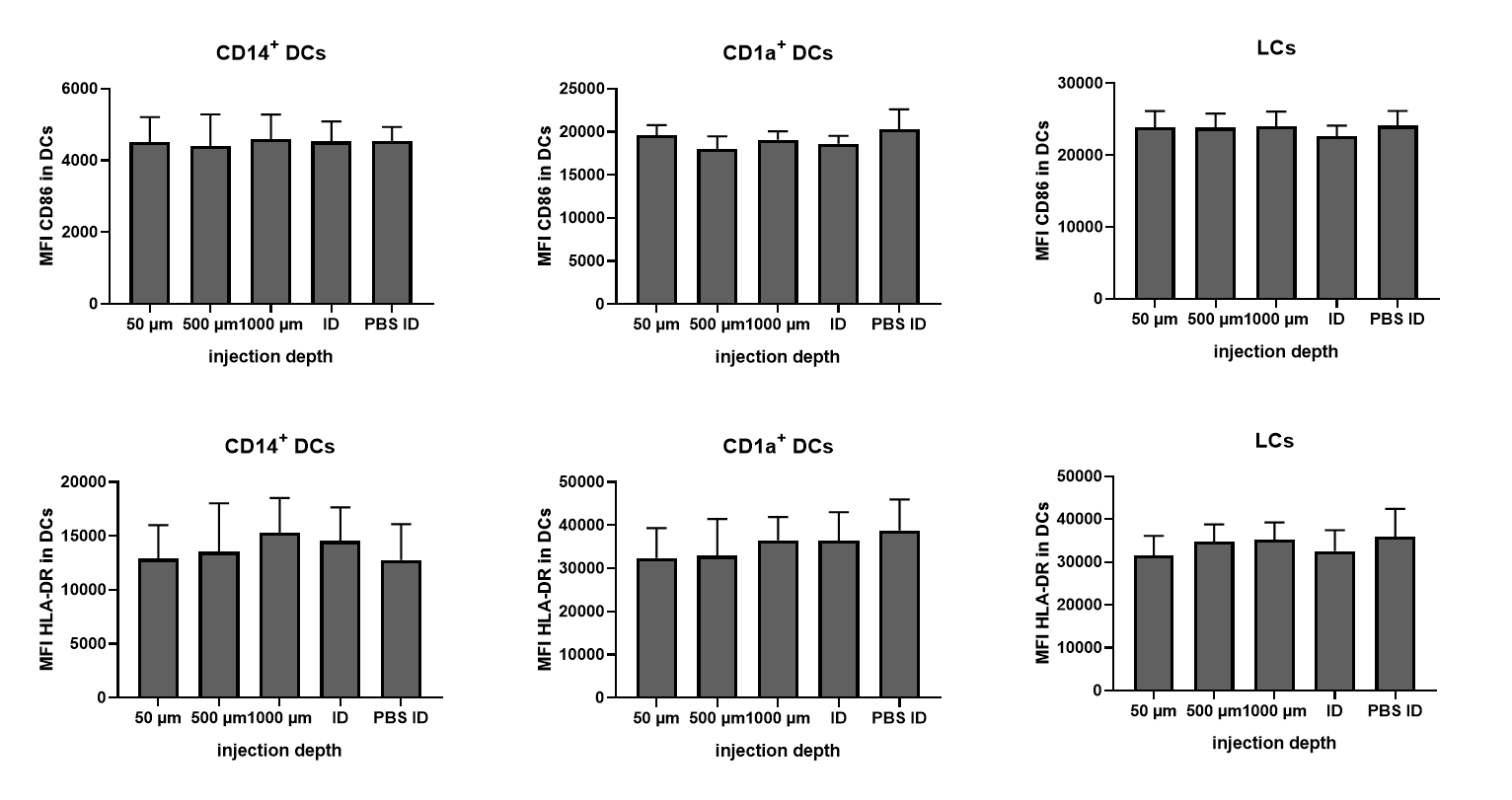


Supplementary Figure 2. Injection depth dependent DC activation. 0.1 µg OVA was injected at different depths in ex vivo human skin explants and compared to conventional intradermal injection. Activation markers CD86 (upper plots) and HLA-DR (lower plots) on different dDC subsets were measured (mean ± SEM (n = 3)).

Supplementary Figure 3. Antigen uptake by DC subsets. Mean fluorescence intensity of the antigen-label in CD14 dDCs, CD1a dDCs and LCs.


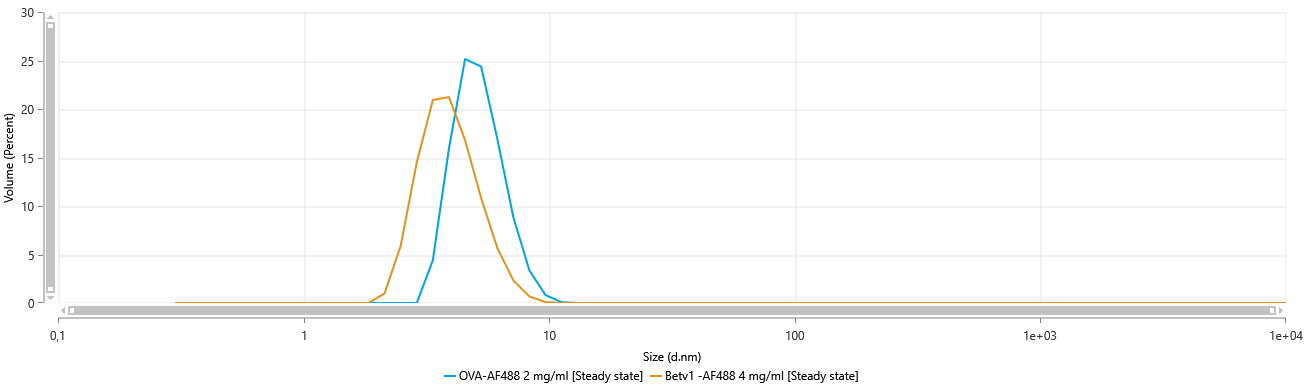


Supplementary Figure 4. Size distribution by volume of OVA-AF488 (blue) and Bet v 1-AF488 (orange) as measured by DLS.


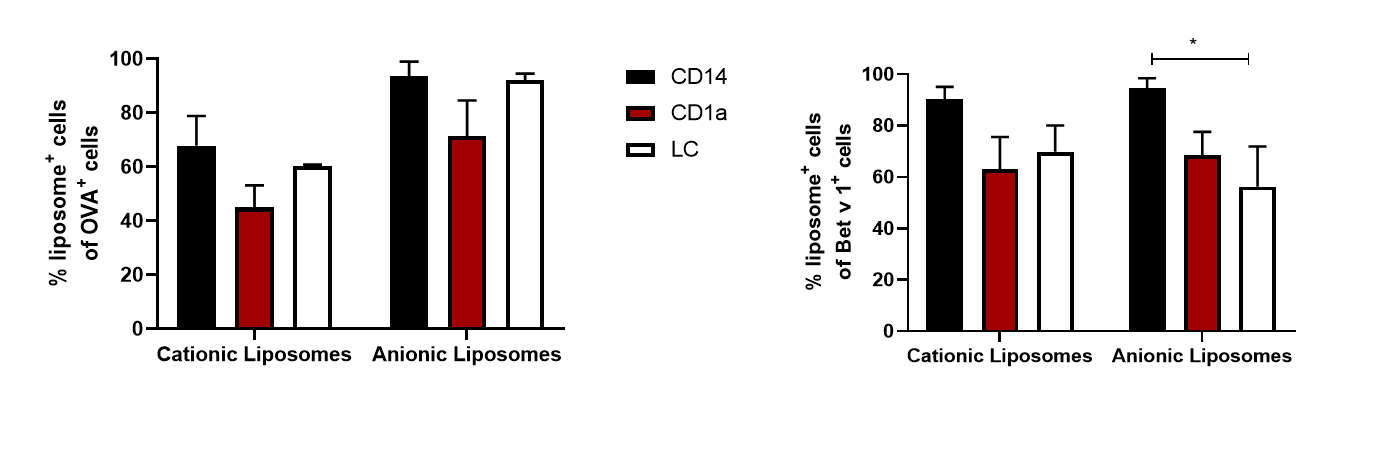


Supplementary Figure 5. Co-uptake of antigen and liposomes. Liposome uptake by dDCs that had also taken up antigen after injection at 500 µm depth in ex vivo human skin explants of OVA-containing formulations (left) and Bet v 1-containing formulations (right) 1 (mean ± SEM; n ≥ 4). The difference between antigens was compared with a 2-way ANOVA. * = p < 0.05.


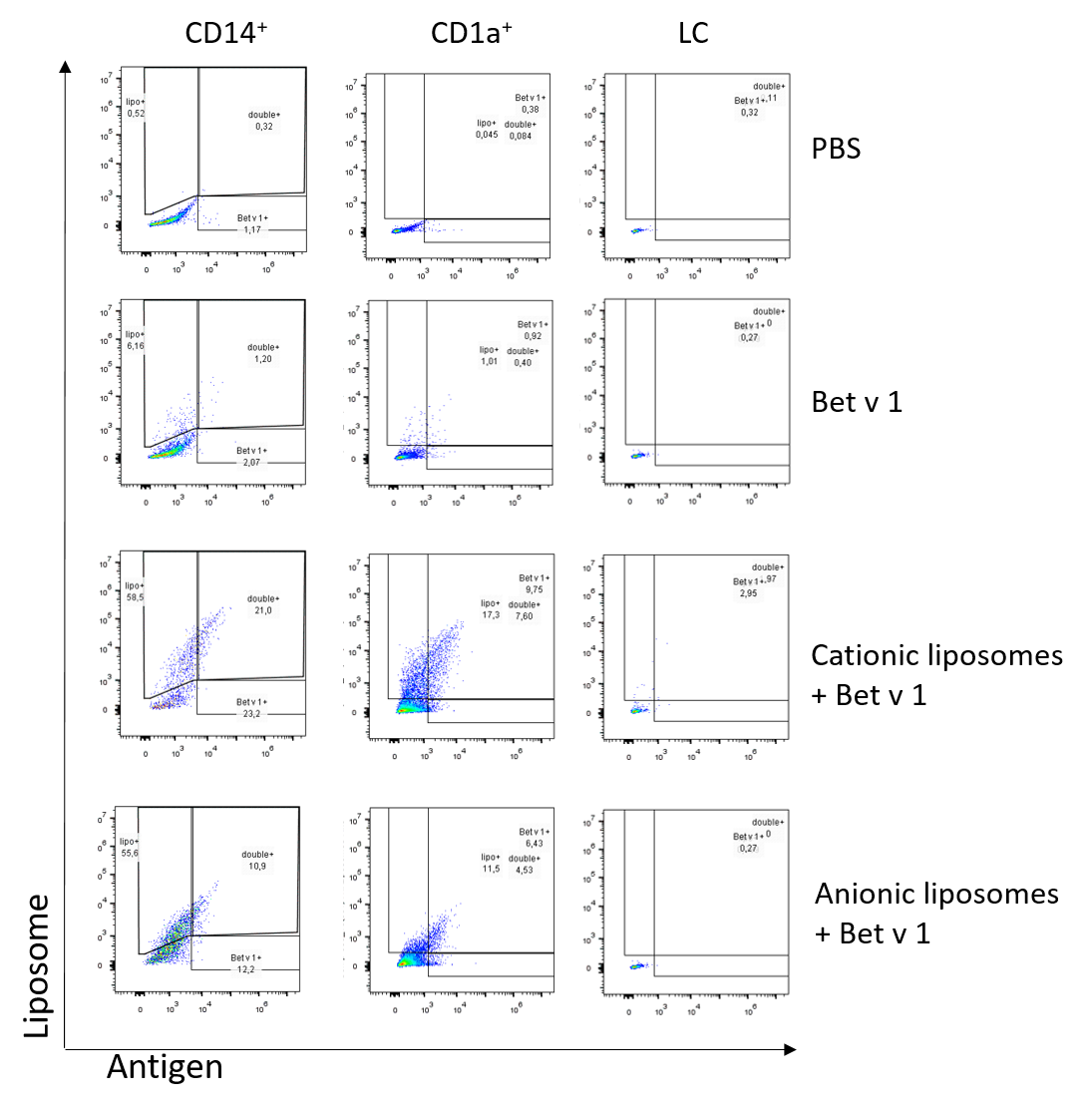


Supplementary Figure 6. Representative flow cytometry plots of uptake of liposomes and Bet v 1 in different APC subsets after injection at 500 µm depth in ex vivo skin explants.


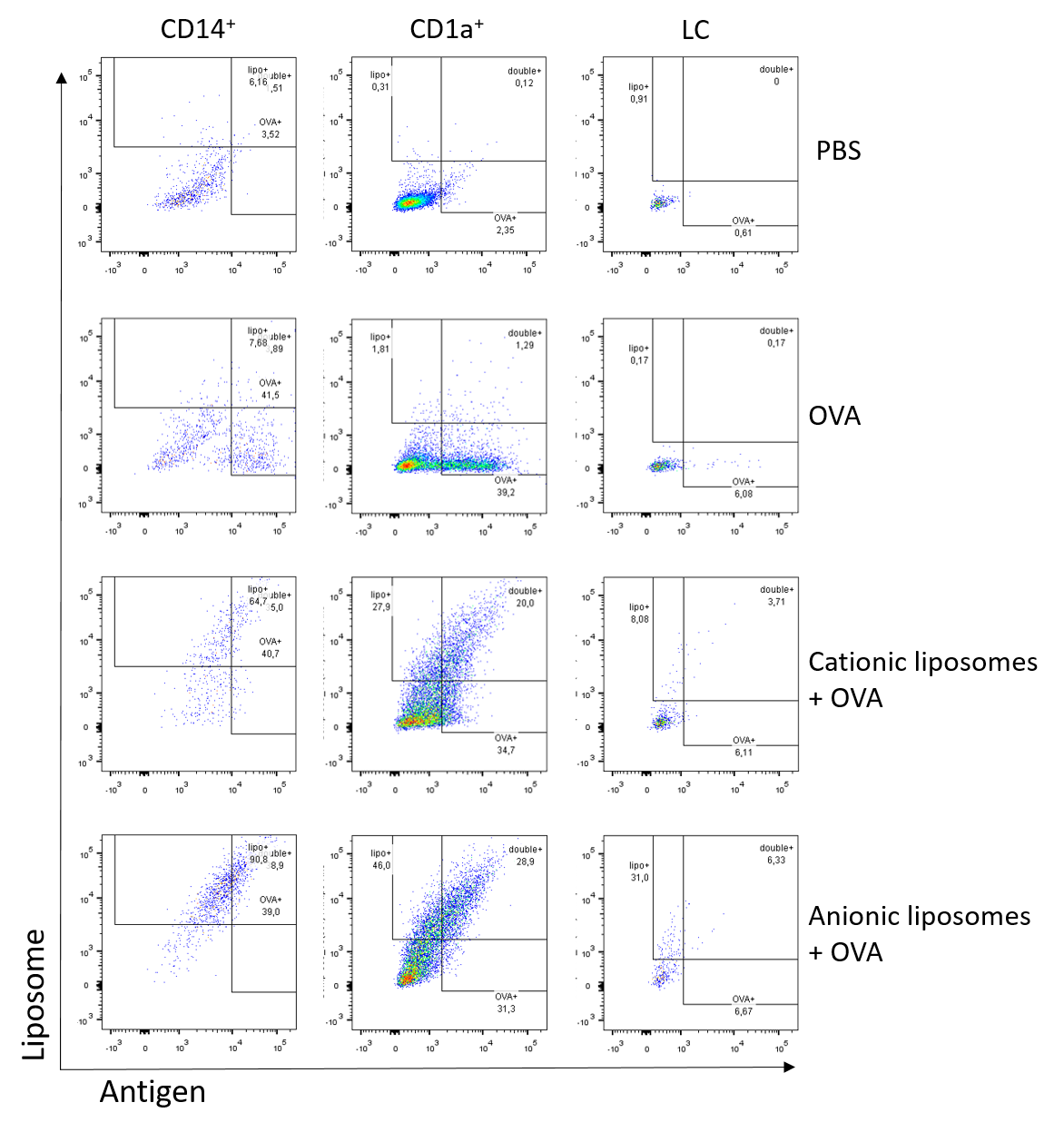


Supplementary Figure 7. Representative flow cytometry plots of uptake of liposomes and OVA in different APC subsets after injection at 500 µm depth in ex vivo skin explants.
